# Neglect scoring modifications in the National Institutes of Health Stroke Scale improve right hemisphere stroke lesion volume prediction

**DOI:** 10.1111/ene.16133

**Published:** 2023-11-17

**Authors:** Adriana Henriques Silva, Pedro Nascimento Alves, Ana Catarina Fonseca, Teresa Pinho‐e‐Melo, Isabel Pavão Martins

**Affiliations:** ^1^ Laboratório de Estudos de Linguagem, Centro de Estudos Egas Moniz, Faculdade de Medicina Universidade de Lisboa Lisboa Portugal; ^2^ Unidade de Acidentes Vasculares Cerebrais, Serviço de Neurologia Hospital de Santa Maria, CHULN Lisboa Portugal; ^3^ Centro de Estudos Egas Moniz, Faculdade de Medicina Universidade de Lisboa Lisboa Portugal; ^4^ Serviço de Neurologia Hospital de Santa Maria, CHULN Lisboa Portugal

**Keywords:** neglect, NIHSS, stroke

## Abstract

**Background:**

The National Institutes of Health Stroke Scale (NIHSS) does not equitably assess stroke severity in the two cerebral hemispheres. By attributing a maximum of two points for neglect and seven for language, it undervalues right hemisphere deficits. We aimed to investigate if NIHSS equally predicts right hemisphere lesion volumes in patients with and without neglect, and if a modification of the neglect scoring rules could increase its predictive capacity.

**Methods:**

We analyzed a prospective cohort of acute right middle cerebral artery ischemic stroke patients. First, we calculated the correlation between NIHSS scores and lesion volume and analyzed the partial correlation of neglect. Then, we applied different modifications in the neglect scoring rules and investigated how they interfered with lesion volume predictive capacity.

**Results:**

A total of 162 ischemic stroke patients were included, 108 with neglect and 54 without. The correlation between lesion volume and NIHSS was lower in patients with neglect (*r* = 0.540 vs. *r* = 0.219, *p* = 0.004) and neglect was a statistically significant covariate in the partial correlation analysis between NIHSS and lesion volume (*p* = 0.017). With the neglect score tripled and with the duplication or triplication of all neglect modalities, the correlation was significantly higher than with the standard NIHSS (*p* = 0.043, *p* = 0.005, *p* = 0.001, respectively). With these modifications, neglect was no longer a significant covariable in the partial correlation between lesion volume and NIHSS.

**Conclusion:**

A modification of NIHSS neglect scoring might improve the scale's capacity to predict lesion volume.

## Abstract 2

In this study, neglect was a statistically significant covariate in the partial correlation analysis between NIHSS and lesion volume. With different modifications of the neglect scoring rules, neglect was no longer a statistically significant factor. These results may increase the NIHSS predictive capacity of the lesion volume.

## INTRODUCTION

The National Institutes of Health Stroke Scale (NIHSS) is a widely used tool for quantifying stroke severity based on neurological impairment scoring [1, 2]. It has an important role in determining the appropriate stroke treatment, namely in selecting patients for intravenous thrombolysis [3, 4] and mechanical thrombectomy [5, 6, 7, 8, 9]. It is also an independent predictor of patients' outcomes [10, 11, 12]. Despite its relevance and broad validation, prior studies suggest that this scale does not equitably assess the two cerebral hemispheres, reporting that the lesion volume in the right hemisphere (RH) is underestimated by NIHSS, compared to left hemisphere (LH) lesions [13, 14]. Patients with RH lesions more frequently present discrepancies between NIHSS and lesion volumes, with low NIHSS but large lesion volumes [14]. More than one‐third of these patients present mild to moderate functional disabilities 3–6 months after stroke [14]. This may happen due to the attribution in the NIHSS of maximum of only two points for neglect [13], a typical sign of RH dysfunction [15]. In comparison, seven points might be attributed to language deficits [13], most commonly associated with LH lesions [15].

Indeed, some studies found that LH strokes are more frequently admitted to acute stroke treatments [16, 17] and have better outcomes [18]. There is also the possibility that RH stroke patients with low NIHSS are being excluded and, therefore, underrepresented in treatment trials [19]. Neglect is associated with longer lengths of stay in rehabilitation units [20, 21, 22, 23], poorer rehabilitation improvements [21, 22, 23, 24, 25], and slower functional progress during rehabilitation [23], when compared to patients without neglect.

The volume of stroke lesions is an independent predictor of stroke patients' long‐term functional status [26, 27, 28]. In this way, a better correlation between lesion volume and NIHSS would increase the capacity of this score to predict stroke outcomes.

Here, we analyzed a group of patients with right middle cerebral artery ischemic stroke, aiming to understand if NIHSS equally predicts RH lesion volumes in patients with and without neglect, and if a modification of the neglect scoring rules could increase its predictive capacity.

## METHODS

### Study sample

We performed a post‐hoc analysis of the RePS (Reduplicative Paramnesia after Stroke) study. RePS was a prospective cohort study that screened 400 consecutive patients with either ischemic or hemorrhagic acute strokes for the presence of spatial delusions. Recruitment lasted from December 2016 to February 2020 and was performed in the Stroke Unit of Santa Maria Hospital, Lisbon, Portugal [29]. Adult patients with RH stroke who were admitted in the acute phase (72 h or less) of stroke were included in RePS. Exclusion criteria were: no lesion or poorly defined lesion in brain imaging; brain images not available; decreased level of consciousness, either somnolence, stupor, or coma; acute confusional state, according to the criteria defined in the *Fifth Edition of the Diagnostic and Statistical Manual of Mental Disorders*; aphasia or severe dysarthria, precluding screening for spatial delusions; and epileptic seizures.

To perform this post‐hoc analysis, we selected patients with a first‐ever ischemic stroke of the right middle cerebral artery territory. Patients that presented previous focal brain lesions in computed tomography (CT) or magnetic resonance imaging (MRI) or that had lesions in other vascular territories were excluded.

The study was approved by the Ethics Committee of the Lisbon Academic Medical Center (No. 395/18) and conducted according to the Declaration of Helsinki. Written informed consent was waived because the study did not imply the performance of specific procedures and all data were anonymized.

### Clinical data

We recorded data about patients' demographics, stroke aetiology (according to the TOAST system) [30], total NIHSS score, individual NIHSS items scores at admission, acute phase treatment (intravenous thrombolysis, mechanical thrombectomy, both or neither), and the imaging modalities available (CT or MRI).

**FIGURE 1:**
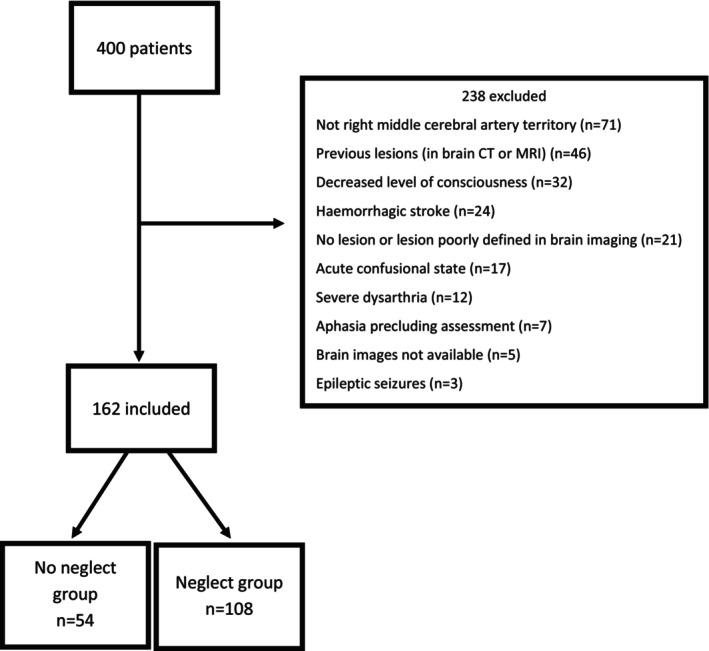
Flowchart of patient selection and classification. CT, computed tomography; MRI, magnetic resonance imaging.

The study sample (162 patients) was divided into two groups: patients without neglect at admission (No Neglect group, *n* = 54) and patients with neglect at admission (Neglect group, *n* = 108 patients). The flowchart of patients' inclusion, exclusion, and classification is illustrated in Figure 1.

The division into ‘Neglect group’ and ‘No Neglect group’ was made according to the NIHSS item 11 (“Extinction/inattention”) at admission. When the patients presented a deterioration of neurological status before acute phase treatment, the NIHSS after the deterioration was considered (*n* = 14).

We then collected the modalities of neglect presented by the patients: visual extinction, tactile extinction, auditory extinction, motor extinction, visuospatial neglect, anosognosia, and anosodiaphoria. The extraction of these data was retrospective, based on the emergency department and stroke unit admission reports.

### Lesion volume quantification

The infarcted areas were manually delimited in the native space using FSLeyes version 0.32.0, on axial slices with a thickness of 3 mm, by a researcher with clinical experience in acute stroke lesion analysis. For patients with MRI (*n* = 57) images available, the diffusion‐weighted imaging (DWI) sequence was the sequence of reference. For the remaining, the lesion was delimited in the CT (*n* = 105) performed 24–72 h after stroke onset. The researcher was blind to patients' clinical status at the time of delimitation. Brain images were normalized to the MNI152 space. For MRI, T1 images were linearly registered to the MNI152 by applying an affine transformation (12 degrees of freedom) using the FSL software FLIRT. Then, a nonlinear registration was conducted using FSL's FNIRT tool [31]. For CT scans, a linear registration was performed to the CT‐derived MNI152 template, by applying an affine transformation (12 degrees of freedom) using FLIRT [32]. This template derived from the alignment of 35 CT scans of healthy individuals with a mean age of 65 years and was specifically designed for the registration of stroke CT images in the MNI152 space [33].

The registration warps were applied to lesion masks. The infarct volume (cm^3^) was calculated using fslstats.

### Correlation between NIHSS and lesion volume

First, we correlated NIHSS scores and lesion volume in the Neglect and in the No Neglect groups. Second, we analyzed: (a) if there was a statistically significant difference between the correlations of the two groups; (b) if the variable group (i.e., presence of neglect) was a statistically significant covariate in the partial correlation analysis between NIHSS and lesion volume. Third, we performed exploratory modifications of the neglect scoring in the NIHSS scale to analyze if they significantly increase the correlation between NIHSS and lesion volume in the Neglect group. The modifications that were performed in the neglect scoring rules were: (a) sum of all neglect modalities documented, each one having a score of one (label: ‘All neglect modalities’; maximum of 7 points); (b) double of the original neglect score (label: ‘Neglect x2’); (c) triple of the original neglect score (label: ‘Neglect x3’); (d) original neglect score raised to the 2nd power (label: ‘Neglect x^2^’); (e) original neglect score raised to the 3rd power (label: ‘Neglect x^3^’); (f) double of the sum of all neglect modalities documented (label: ‘All neglect modalities x2’); and (g) triple of the sum of all neglect modalities documented (label: ‘All neglect modalities x3’).

For each modification, we subtracted the original neglect score from the NIHSS score and added the modified neglect score. Then, we calculated the correlation between each modified NIHSS with lesion volume and the partial correlation between lesion volume, NIHSS, and the variable group (i.e., presence of neglect) and we analyzed if there was a statistically significant difference between the original correlation and the correlation obtained with the NIHSS score modification.

The performance of recanalization therapies may influence the measured lesion volumes. Therefore, we also performed a sensitivity analysis including only the patients submitted to recanalization therapies (recombinant tissue plasminogen activator [rtPA] and/or endovascular thrombectomy [EVT]).

### Statistical analysis

Baseline features of the patients were compared in both groups. Continuous variables were compared with unpaired Student's *t*‐test or Mann–Whitney test, according to their distribution. Categorical variables were compared using Chi‐square or Fisher test, as appropriate.

Spearman correlation was used to determine the strength of association between the NIHSS and lesion volumes. The statistical difference between correlations was calculated using the R package ‘cocor’ (http://comparingcorrelations.org).

All statistical analyses were performed using SPSS Version 26. An alpha value of 0.05 was set for statistical significance. To correct for multiple comparisons, a Bonferroni correction was applied.

## RESULTS

### Demographic and clinical features

The patients' demographic and clinical features are summarized in Table 1. Patients with neglect had higher NIHSS scores at admission, larger lesion volumes, and had different aetiology and treatment distributions. No statistical differences were found in age and imaging modality of reference.

**TABLE 1:** Patients’ baseline features.

Feature	No Neglect group (*n*=54 patients)	Neglect group (*n*=108 patients)	*P*‐value
Age (years), mean ± SD	66.11 ± 13.33	70.05 ± 13.36	0.079
NIHSS at admission, mean ± SD	9.52 ± 5.89	14.06 ± 4.81	<0.001
Volume (cm^3^), median [IQR]	6942.50 [1820.75; 31787.25]	37209.50 [10381.25; 96357.50]	<0.001
Etiology, *n* (%)			0.025
Large artery atherosclerosis	10 (18.5)	10 (9.3)	
Cardioembolism	19 (35.2)	58 (53.7)	
Small‐vessel occlusion	2 (3.7)	0 (0)	
Other determined	1 (1.9)	6 (5.6)	
Undetermined	22 (40.7)	34 (31.5)	
Imaging, *n* (%)			0.081
CT	30 (55.6)	75 (69.4)	
MRI	24 (44.4)	33 (30.6)	
Time between stroke and imaging (days), median [IQR]			
CT	2 [1;2]	1 [1;2]	0. 230
MRI	2 [1;4]	3 [2;5]	0.314
Treatment, *n* (%)			<0.001
None	15 (27.8)	5 (4.6)	
rtPA	16 (29.6)	31 (28.7)	
EVT	14 (25.9)	27 (25.0)	
rtPA+EVT	9 (16.7)	45 (41.7)	

Abbreviations: CT, computed tomography; EVT, endovascular thrombectomy; IQR, interquartile range; MRI, magnetic resonance imaging; NIHSS, National Institutes of Health Stroke Scale; rtPA, recombinant tissue plasminogen activator; SD, standard deviation.

The frequencies of the different modalities of neglect are presented in Table 2.

**TABLE 2:** Frequencies of the different modalities of neglect. In three patients the type of neglect was not characterized (their neglect score in National Institutes of Health Stroke Scale was two).

Type of neglect	*n* (%)
Anosognosia	73 (67.6)
Anosodiaphoria	1 (0.9)
Visual extinction	15 (13.9)
Auditory extinction	36 (33.3)
Motor extinction	4 (3.7)
Visuospatial neglect	5 (4.6)
Tactile extinction	43 (39.8)

The mean of the sum of all neglect modalities was 1.68 ± 0.76 and the median was 2 [1.53;1.82].

### Correlation between NIHSS and lesion volume

**FIGURE 2:**
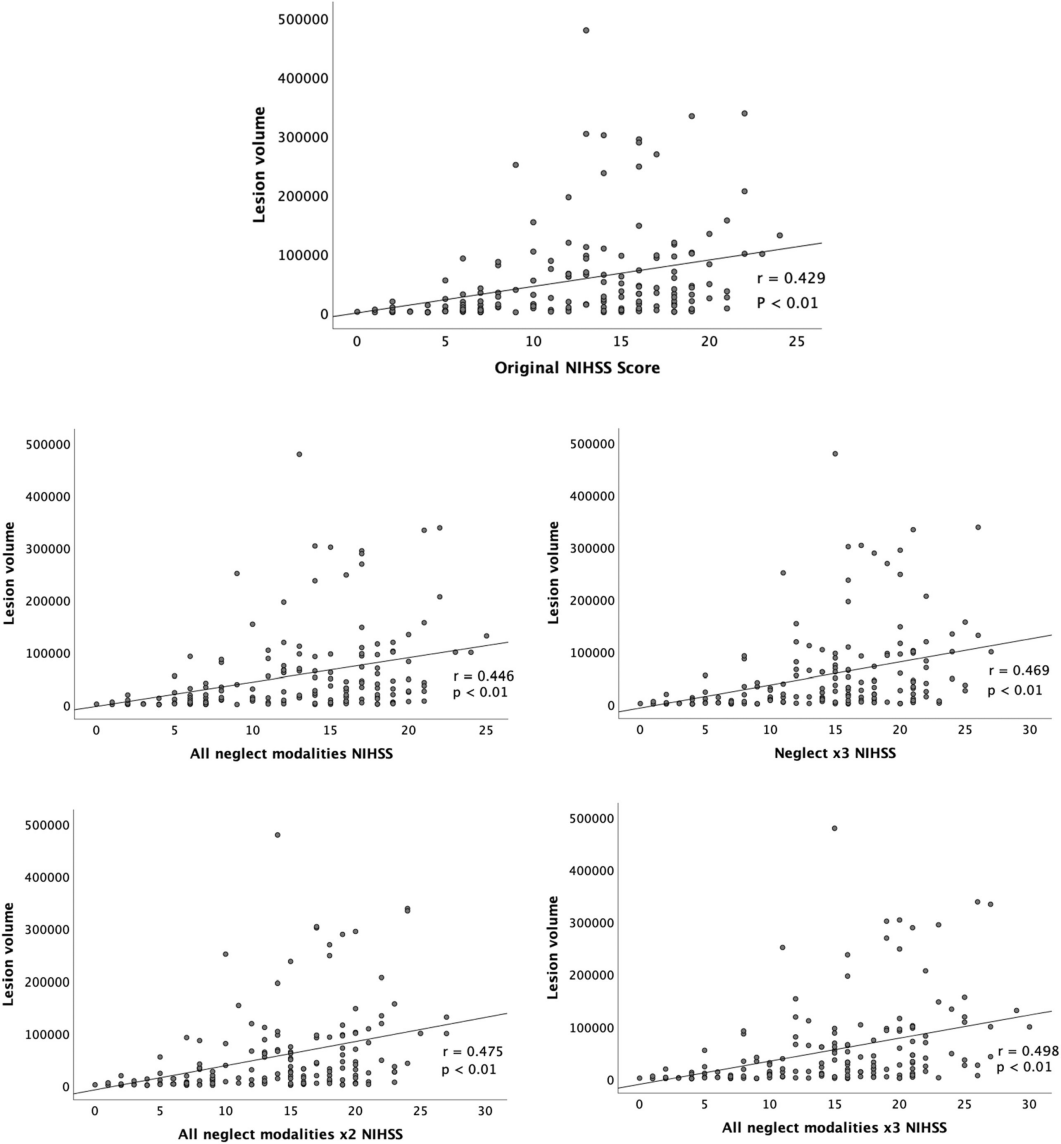
National Institutes of Stroke Scale (NIHSS) score and lesion volume. *r*, Spearman correlation between NIHSS and lesion volume; *p*, *p*‐value; Neglect x3 NIHSS, NIHSS with the original neglect score tripled; All neglect modalities NIHSS, NIHSS with the sum of all neglect modalities; All neglect modalities x2 NIHSS, NIHSS with the sum of all neglect modalities doubled; All neglect modalities x3 NIHSS, NIHSS with the sum of all neglect modalities tripled.

The Spearman correlation between lesion volume and original NIHSS was *r* = 0.429 (*p* < 0.01; Table 3, Figure 2).

**TABLE 3:** Spearman correlation between lesion volume and National Institutes of Stroke Scale (NIHSS), partial correlation between lesion volume, NIHSS, and the variable group (i.e., presence of neglect) and the statistical difference between original NIHSS correlation and modified NIHSS correlation.

Parameter	Spearman correlation between lesion volume and NIHSS	Partial correlation between lesion volume and the variable group controlling for NIHSS	Statistical difference between original NIHSS correlation and modified NIHSS correlation
Original NIHSS	0.429 (*p* < 0.01)	0.188 (*p* = 0.017)	
All neglect modalities NIHSS	0.446 (*p* < 0.01)	0.178 (*p* = 0.024)	*p* = 0.008
Neglect x2 NIHSS	0.446 (*p* < 0.01)	0.154 (*p* = 0.051)	*p* = 0.0586
Neglect x3 NIHSS	0.464 (*p* < 0.01)	0.124 (*p* = 0.119)	*p* = 0.043
Neglect x^2^ NIHSS	0.443 (*p* < 0.01)	0.170 (*p* = 0.031)	*p* = 0.2203
Neglect x^3^ NIHSS	0.456 (*p* < 0.01)	0.152 (*p* = 0.054)	*p* = 0.3669
All neglect modalities x2 NIHSS	0.475 (*p* < 0.01)	0.133 (*p* = 0.092)	*p* = 0.005
All neglect modalities x3 NIHSS	0.498 (*p* < 0.01)	0.095 (*p* = 0.233)	*p* = 0.006

There was a statistically significant difference between the correlation of lesion volume and NIHSS score in the No Neglect group and Neglect group (*r* = 0.540 and *r* = 0.249, respectively; *p* = 0.04).

The partial correlation between group and volume, after controlling for NIHSS, was statistically significant (*r* = 0.188, *p* = 0.017).

Spearman correlation between NIHSS and lesion volume and the partial correlation between group and volume in the different modied NIHSS are presented in Table 3.

The partial correlation between group and lesion volume, after controlling for NIHSS, was not statistically significant in the following NIHSS modifications: Neglect x2 NIHSS, Neglect x3 NIHSS, Neglect x^3^ NIHSS, All neglect modalities x2 NIHSS, All neglect modalities x3 NIHSS (Table 3).

Spearman correlation between modified NIHSS and lesion volume was significantly higher than the correlation obtained with the original NIHSS in the following modifications: All neglect modalities NIHSS, Neglect x3 NIHSS, All neglect modalities x2 NIHSS, and All neglect modalities x3 NIHSS (Figure 2; Table 3).

With the duplication or triplication of all neglect modalities NIHSS, the correlation was also significantly higher with Bonferroni correction for multiple comparisons (*p* = 0.007).

### Correlation between NIHSS and lesion volume including only the patients submitted to recanalization therapies

The results of the sensitivity analysis, including only the patients submitted to recanalization therapies (rtPA and/or EVT), were similar. The partial correlation between group and volume, after controlling for NIHSS, was statistically significant (*r* = 0.179, *p* = 0.033). The partial correlation between group and lesion volume, after controlling for NIHSS, was not statistically significant in the following NIHSS modifications: Neglect x2 NIHSS, Neglect x3 NIHSS, Neglect x^2^ NIHSS, Neglect x^3^ NIHSS, All neglect modalities x2 NIHSS, and All neglect modalities x3 NIHSS. In these modifications, the Spearman correlation between modified NIHSS and lesion volume was significantly higher than the correlation obtained with the original NIHSS in All neglect modalities x2 NIHSS and All neglect modalities x3 NIHSS. Spearman correlation between Neglect x3 NIHSS and lesion volume was not significantly higher than the correlation obtained with the original NIHSS; however, there was a tendency towards statistical significance (*p* = 0.054) (Table S1).

## DISCUSSION

In this study, we investigated the relationship between lesion volume and NIHSS in patients with ischemic stroke of the right middle cerebral artery with and without neglect. First, we found a significant correlation between lesion volume and NIHSS score as reported in previous studies [13, 14]. Second, we showed that the correlation between lesion volume and NIHSS was lower in patients with neglect, and that neglect was a statistically significant factor for this correlation. Finally, we disclosed that modifications of neglect scoring in the NIHSS significantly improved the correlation between lesions volume and NIHSS. This effect was obtained by tripling the original NIHSS neglect score, by doubling the sum of all neglect modalities, or by tripling the sum of all neglect modalities.

Neglect can be one of the most disabling consequences of a stroke [21, 22, 23], and negatively affects the activities of daily living [25]. There are different modalities of neglect – visual, tactile, auditory, motor, and visuospatial – and also neglect‐related disorders such as anosognosia and anosodiaphoria [34, 35, 36]. There is not a single test that identifies all modalities of neglect [37, 38, 39]. Neglect is more common in cardioembolic than in non‐cardioembolic stroke, as we also observed in our study. This association might be driven by the larger infarct volumes and by the more frequent cortical involvement of cardioembolic strokes [40]. In this study, all the modifications in neglect scoring rules showed a tendency to increase the correlation between NIHSS and lesion volume. However, only the correlations between lesion volume and NIHSS with the sum of all neglect modalities, with the neglect score tripled, with the sum of all neglect modalities doubled, and with the sum of all neglect modalities tripled were statistically higher. In this way, our analysis suggests that these four modifications may improve the prediction of lesion volume. In these four modifications, only the partial correlation between lesion volume and the variable group, controlling for all neglect modalities NIHSS, remained statically significant. This may be due to the fact that not all neglect modalities are evaluated at admission.

It has been reported that for the same lesion volume the mean NIHSS is higher in the LH stroke than in the RH and, consequently, NIHSS underestimates the severity of RH stroke [13, 14]. Increasing the number of points attributed to neglect and making neglect no longer an independent factor for lesion volume prediction, our findings may have implications for clinical practice as they may improve NIHSS volume prediction in the RH.

Based on our results, a rapid assessment of all neglect modalities in the admission time may lead to a more rigorous neglect score, as we already have in the NIHSS language score. Consequently, these results may minimize the lateralization bias between right and left hemisphere.

As for the limitations of our study, it is a post‐hoc analysis of a study not primarily designed to assess neglect, and a detailed neglect testing can be difficult to execute in an acute setting. In our work, complete neglect characterization was described in the admission reports in 86 patients (*n* = 80%). In the remaining patients, some neglect modalities may have not been assessed or registered. In addition, although some studies have found a correlation between lesion volume and stroke severity [27, 28], severity and functional outcome also depend on lesion location [41]. Third, lesion volumes could not be measured before revascularization therapies because pre‐treatment MRI was not performed. Revascularization treatments might introduce bias by reducing lesion volumes. The sensitivity analysis including only patients submitted to recanalization therapies (rtPA and/or EVT) showed similar results. Fourth, we used two different imaging modalities (CT and MRI). Although modern CT scanners provide high‐resolution images suitable for lesion mapping and there was no statistically significant difference in the percentage of patients undergoing CT or MRI between the two groups, CT scans may be less accurate in revealing ischemic regions than MRI. Patients with aphasia or severe dysarthria were not included, which might be a bias, although the number of excluded patients was small (*n* = 19.5%). Finally, we did not have functional outcomes to assess. Their measurement in future studies, besides lesion volume, is needed.

In conclusion, increasing the number of points attributed to neglect, particularly tripling neglect score, doubling the sum of all neglect modalities and tripling the sum of all neglect modalities, may improve the correlation between lesion volume and NIHSS. These results may foster the refinement of neglect scoring in the NIHSS scale, improve patients' selection for recanalization treatments and attenuate the discrepant scoring approaches between neglect and aphasia.

## AUTHOR CONTRIBUTIONS

**Adriana Henriques Silva:** Formal analysis; writing – original draft; investigation; methodology. **Pedro Nascimento Alves:** Methodology; conceptualization; investigation; writing – review and editing; formal analysis. **Ana Catarina Fonseca:** validation; supervision; writing – review and editing. **Teresa Pinho‐e‐Melo:** validation; supervision; writing – review and editing. **Isabel Pavão Martins:** Writing – review and editing; validation; supervision.

## CONFLICT OF INTEREST STATEMENT

The authors declare that they have no conflicts of interest.

## Supporting information


Table S1

## Data Availability

The data that support the findings of this study are available on request from the corresponding author. The data are not publicly available due to privacy or ethical restrictions.
