# Supplementary material for: Neglect scoring modifications in the National Institutes of Health Stroke Scale improve right hemisphere stroke lesion volume prediction
Source: Eur J Neurol. 2023 Nov 17;31(2):e16133. doi: 10.1111/ene.16133 (PMC11235761; doi:10.1111/ene.16133)
Supplement: Supplementary file 1 — Table S1 [file ENE-31-e16133-s001.docx]

Supplementary Table 1

|  | **Spearman correlation between lesion volume and NIHSS** | **Partial correlation between lesion volume and the variable group controlling for NIHSS** | **The statistical difference between original NIHSS correlation and modified NIHSS correlation.** |
| --- | --- | --- | --- |
| **Original NIHSS** | 0.349  (p<0.01) | 0.179  (p=0.033) |  |
| **All neglect modalities NIHSS** | 0.370  (p<0.01) | 0.170  (p=0.043) | p=0.015 |
| **Neglect x2 NIHSS** | 0.370  (p<0.01) | 0.146  (p=0.083) | p=0.059 |
| **Neglect x3 NIHSS** | 0.390  (p<0.01) | 0.115  (p=0.173) | p=0.054 |
| **Neglect x^2^NIHSS** | 0.365  (p<0.01) | 0.162  (p=0.055) | p=0.254 |
| **Neglect x^3^ NIHSS** | 0.375  (p<0.01) | 0.143  (p=0.091) | p=0.882 |
| **All neglect modalities x2 NIHSS** | 0.404  (p<0.01) | 0.128  (p=0.131) | p=0.009 |
| **All neglect modalities x3 NIHSS** | 0.430  (p<0.01) | 0.090  (p=0.291) | p=0.011 |

Supplementary Table 1 - Statistical analysis including only the patients submitted to recanalization therapies - Spearman correlation between lesion volume and NIHSS, Partial correlation between lesion volume, NIHSS and the variable group (i.e. presence of neglect) and the statistical difference between original NIHSS correlation and modified NIHSS correlation.
